# Risk factors associated with postoperative lymphocele in patients with gynecological malignant tumors: a systematic review and meta-analysis

**DOI:** 10.3389/fonc.2026.1828153

**Published:** 2026-06-10

**Authors:** Ting-yu Zhao, Wen Jiang, Jia-min Liu, Zhi-yi Liu, Ping Xie

**Affiliations:** 1 Clinical Medical College, Chengdu University of Traditional Chinese Medicine, Chengdu, China; 2 Department of Traditional Chinese Medicine, West China Second Hospital of Sichuan University, Chengdu, China; 3 Department of Gynecology, Hospital of Chengdu University of Traditional Chinese Medicine, Chengdu, China

**Keywords:** gynecological malignancies, lymphadenectomy, lymphocele, meta-analysis, preventive measures, related factors

## Abstract

**Background:**

Pelvic lymphoceles are a common complication after lymph node dissection for gynecological malignancies, with severe cases leading to significant morbidity and delays in adjuvant therapy. This meta-analysis aims to identify risk factors for postoperative lymphoceles and provide evidence for clinical prevention and management.

**Methods:**

A systematic search was performed across eight databases (CNKI, Wanfang, VIP, CBM, Web of Science, PubMed, Cochrane Library, Embase) from inception to August 15, 2025, to include cohort and case-control studies. Two investigators independently screened studies and extracted data. Methodological quality was assessed using the Newcastle-Ottawa Scale, and statistical analyses were conducted with STATA 18.0. Meta-analysis employed fixed- or random-effects models, with heterogeneity evaluated by the I² statistic. The study was registered in PROSPERO (CRD420251053680).

**Results:**

64 studies involving 15,739 patients were included. The pooled incidence of postoperative lymphocele was 30.18%. Moderate to high between-study heterogeneity was detected for most outcomes. Key significant risk factors were as follows: age >50 years (OR = 1.21, 95%CI: 1.02–1.42), BMI ≥24 kg/m² (OR = 1.45, 95%CI: 1.10–1.93), diabetes mellitus (OR = 1.30, 95%CI: 1.11–1.52), laparotomy (OR = 2.76, 95%CI: 2.12–3.58), resection of >20 lymph nodes (OR = 2.75, 95%CI: 2.16–3.51), pelvic and para-aortic lymphadenectomy (OR = 1.86, 95%CI: 1.42–2.43), omentectomy (OR = 1.56, 95%CI: 1.18-2.07), retroperitoneal closure (OR = 2.44, 95%CI: 1.67–3.57), monopolar electrosurgery (OR = 2.48, 95%CI: 1.75–3.51), prolonged operation time (>3h) (OR = 1.56, 95%CI: 1.10-2.21), 24h drainage >100 ml (OR = 1.61, 95%CI: 1.10-2.36), prolonged drainage (>3d) (OR = 1.60, 95%CI: 1.03-2.46), transvaginal drainage (OR = 2.90, 95%CI: 1.92–4.40), ovarian cancer (OR = 1.69, 95%CI: 1.21–2.36), lymphovascular invasion (OR = 1.25, 95%CI: 1.03–1.52), deep myometrial invasion (OR = 1.89, 95%CI: 1.25–2.87), lymph node positivity (OR = 1.59, 95%CI: 1.26–2.00), anemia (OR = 1.20, 95%CI: 1.02–1.40), hypoalbuminemia (OR = 1.58, 95%CI: 1.07–2.34), postoperative chemotherapy (OR = 1.85, 95%CI: 1.29–2.65), and concurrent chemoradiotherapy (OR = 2.49, 95%CI: 1.68–3.69). Advanced FIGO stage was also significant.

**Conclusions:**

Clinicians can stratify lymphocele risk by integrating preoperative features, surgical procedures, tumor pathology, nutritional status, and adjuvant therapy to guide individualized management. Intraoperatively, rational planning, limited lymphadenectomy, retroperitoneal preservation, and standardized use of energy devices are recommended. Postoperatively, optimized drainage and correction of nutritional disorders reduce lymphocele risk. Given heterogeneity in some risk factors and predominance of single-center retrospective studies, well-designed prospective studies with unified criteria are needed to validate and refine prevention strategies.

**Systematic review registration:**

https://www.crd.york.ac.uk/prospero/, identifier PROSPERO (CRD420251053680).

## 1 Introduction

Surgery is the core therapeutic modality for gynecological malignancies such as endometrial cancer, ovarian cancer, and early-stage cervical cancer. Pelvic and para-aortic lymphadenectomy constitutes an indispensable part of tumor staging and clinical management. Pelvic lymphocele is one of the most common complications following lymph node dissection (1–3), with an incidence of 23% to 63% as reported in the literature (4). Most lymphoceles are asymptomatic and can be spontaneously absorbed (3, 5); however, symptomatic or infected lymphoceles may give rise to abdominal pain, lower extremity edema, deep vein thrombosis, hydronephrosis, and other complications, which severely compromise patients’ quality of life (1, 3, 6). Moreover, such conditions may further lead to severe adverse events including infection and sepsis, and even delay the administration of postoperative adjuvant therapy, thereby affecting tumor prognosis (7–9).

Existing studies have indicated that the formation of lymphoceles is mainly associated with intraoperative injury to lymphatic vessels and lymph nodes, which results in lymphatic drainage disorders and subsequent accumulation of lymph fluid in the retroperitoneal space (10). Despite the growing number of studies on the risk factors for lymphoceles, most of these studies adopt small-sample and single-center designs. The results of these studies are markedly inconsistent (2), which may stem from variations in diagnostic and therapeutic strategies, study designs, detection modalities, and other confounding factors, leading to a lack of reliable and unified evidence-based basis. Meanwhile, most existing studies have failed to systematically summarize multi-dimensional perioperative factors, and high-level evidence provided by large-sample Meta-analyses is still insufficient.

Therefore, this study systematically searched relevant cohort studies and case-control studies at home and abroad, and conducted a comprehensive Meta-analysis from five aspects: preoperative characteristics, surgery-related factors, tumor pathology, postoperative nutritional and metabolic status, and perioperative adjuvant therapy. The purpose of this study is to clarify the key risk factors for lymphoceles after gynecological malignancy surgery, so as to provide evidence-based support for the identification of clinical high-risk populations, the optimization of perioperative management, and the prevention of complications.

## 2 Materials and methods

This systematic review and meta-analysis were conducted and reported in accordance with the current Preferred Reporting Items for Systematic Reviews and Meta-Analyses (PRISMA) guidelines (11). The protocol has been registered with the International Prospective Systematic Reviews Register (PROSPERO) under number CRD420251053680.

### 2.1 Search strategy

A comprehensive literature search was conducted across eight electronic databases, including four Chinese databases (China National Knowledge Infrastructure [CNKI], Wanfang Data, VIP Chinese Science and Technology Journal Database, and China Biomedical Literature Service System [SinoMed]) and four English databases (Web of Science, PubMed, Cochrane Library, and Embase). The search covered all publications from the inception of each database to August 15, 2025.

To ensure comprehensiveness and precision, database-specific search strategies were formulated using a combination of controlled vocabulary (subject headings) and free-text terms, tailored to the indexing system of each platform. The core concepts included “gynecological malignancies”, “lymphadenectomy”, “lymphocele”,” and “related factors.” Boolean operators (AND, OR) were systematically employed to combine these terms into a structured search query. Manual retrieval was further supplemented by screening the reference lists of eligible studies, with primary attention paid to original articles published in Chinese and English due to language accessibility and data extractability.

### 2.2 Eligibility criteria

Studies were included if they met the following criteria: (i) Study design: cohort studies or case-control studies, restricted to publications in Chinese or English only; (ii) Study population: patients pathologically diagnosed with gynecological malignancies (including cervical cancer, endometrial cancer, and ovarian cancer) who underwent lymphadenectomy; (iii) Diagnostic criteria for lymphocele: lymphocele confirmed by ultrasound, CT, or MRI; (iv) Outcome measures: risk factors or associated factors for postoperative lymphocele, with reported OR values and 95% confidence intervals (95%CI) either directly or indirectly.

Studies were excluded if they met the following exclusion criteria: (i) published in languages other than Chinese or English; (ii) duplicate publications; (iii) conference abstracts, reviews, case reports, systematic reviews, meta-analyses, or unpublished literatures; (iv) full texts unavailable; (v) insufficient, unconvertible or redundant key data; or (vi) low methodological quality (Newcastle–Ottawa Scale score < 4).

### 2.3 Literature screening and data extraction

Two researchers independently completed literature screening and data extraction. Any discrepancies were resolved through cross-checking and further consultation with a third senior investigator until a consistent conclusion was reached. All retrieved literatures were imported into Zotero software to remove duplicate records. Initial screening was performed by reviewing titles and abstracts to exclude irrelevant studies. The remaining potential literatures were further assessed via full-text review in strict accordance with the predefined eligibility criteria.

A standardized Excel data extraction form was adopted to collect basic information, including article title, first author, publication date, country, study design, tumor type, sample size, imaging diagnostic methods, follow-up duration, diagnostic timing, and relevant data of lymphocele risk factors. For dichotomous variables (e.g., history of diabetes), odds ratios (ORs) with 95% confidence intervals (CIs) were recorded or calculated from total case numbers and event rates.

### 2.4 Literature quality assessment

Two investigators independently evaluated the methodological quality of included cohort and case-control studies using the Newcastle-Ottawa Scale (NOS) (12). Disagreements in quality evaluation were settled through group discussion with a third reviewer. The NOS contains independent evaluation versions for cohort and case-control studies, covering three core domains: group selection, inter-group comparability, and exposure or outcome assessment. The total NOS score ranges from 0 to 9 points, and studies were divided into low quality (0–4 points), moderate quality (5–7 points), and high quality (8–9 points) (13). All included studies in this analysis scored 5–8 points, indicating overall moderate to high methodological quality. Studies with NOS scores ≤4 were ultimately excluded.

### 2.5 Statistical analysis

All statistical analyses were performed using STATA 18.0, with a two-tailed test level set at α = 0.05. Heterogeneity across included studies was evaluated by Cochran’s Q test and I² statistic. A fixed-effects model was adopted when Q test P > 0.10 and I² < 50%, indicating low to moderate heterogeneity; otherwise, a random-effects model was used. For dichotomous outcomes, pooled effect sizes were presented as odds ratios (ORs) with 95% CIs.

Sensitivity analyses were performed by omitting individual studies one by one and switching statistical models to verify the stability and reliability of pooled results. For outcomes incorporating no fewer than 10 studies, funnel plots combined with Egger’s test were applied to assess potential publication bias, and P > 0.05 in Egger’s test was regarded as no significant publication bias (14). Except for special instructions, P < 0.05 was considered statistically significant.

## 3 Results

### 3.1 Literature search results and process

**Figure 1:**
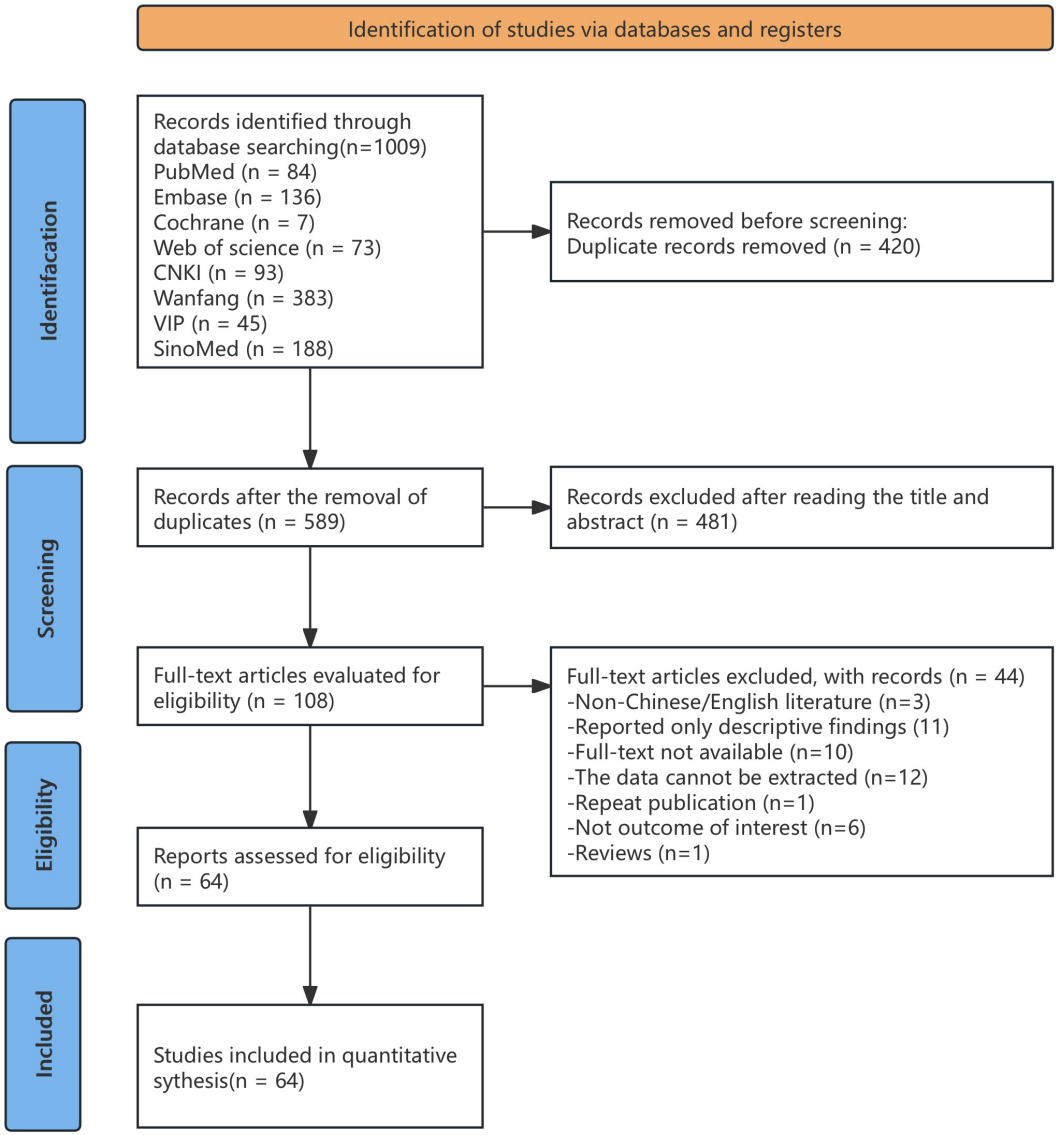
PRISMA flow diagram of the literature screening process.

A total of 1009 records were initially identified through database searching and manual retrieval. After importing into Zotero reference management software, 420 duplicate publications were removed. Following title and abstract screening, 481 records were excluded. Ultimately, 64 studies were included after full-text assessment. The study selection process is detailed in the PRISMA flow diagram (**Figure 1**).

### 3.2 Study characteristics and quality assessment

**Figure 2:**
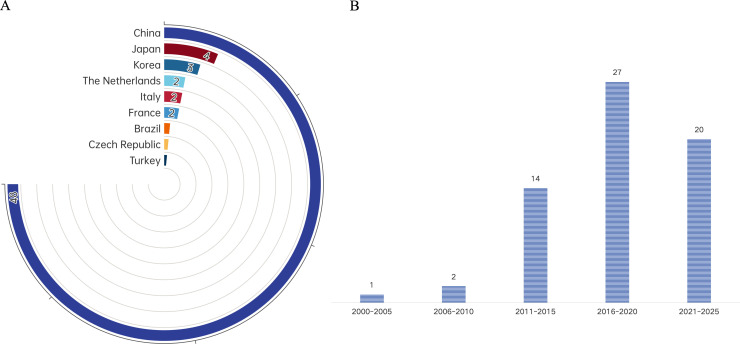
Summary of study characteristics. **(A)** Distribution of publications by country. **(B)** Temporal trend of included studies.

The meta-analysis incorporated 64 eligible studies (15–78), consisting of 6 case–control (23, 32, 48, 61, 68, 74) and 58 cohort studies. Overall, 18 articles were published in English (15–32) and 46 in Chinese (33–78), with most investigations conducted in Asia (48/64), and a small number of studies from Europe and South America (**Figure 2A**). The included literature was issued from 2004 to 2025, and over three-quarters were published in the latest decade (**Figure 2B**), reflecting rising research attention on postoperative lymphocele. The follow-up duration varied substantially across eligible studies, ranging from 2 weeks to 10 years, with an average follow-up time of 13 months. Meanwhile, the time to initial lymphocele diagnosis also differed markedly, spanning from 5 days to 22.3 months. According to the NOS quality assessment, three studies were rated as moderate quality (20, 22, 25), while the remaining 61 included studies achieved high methodological quality (15–19, 21, 23, 24, 26–78).

### 3.3 Meta-analysis

#### 3.3.1 Lymphocele incidence rate

**Figure 3:**
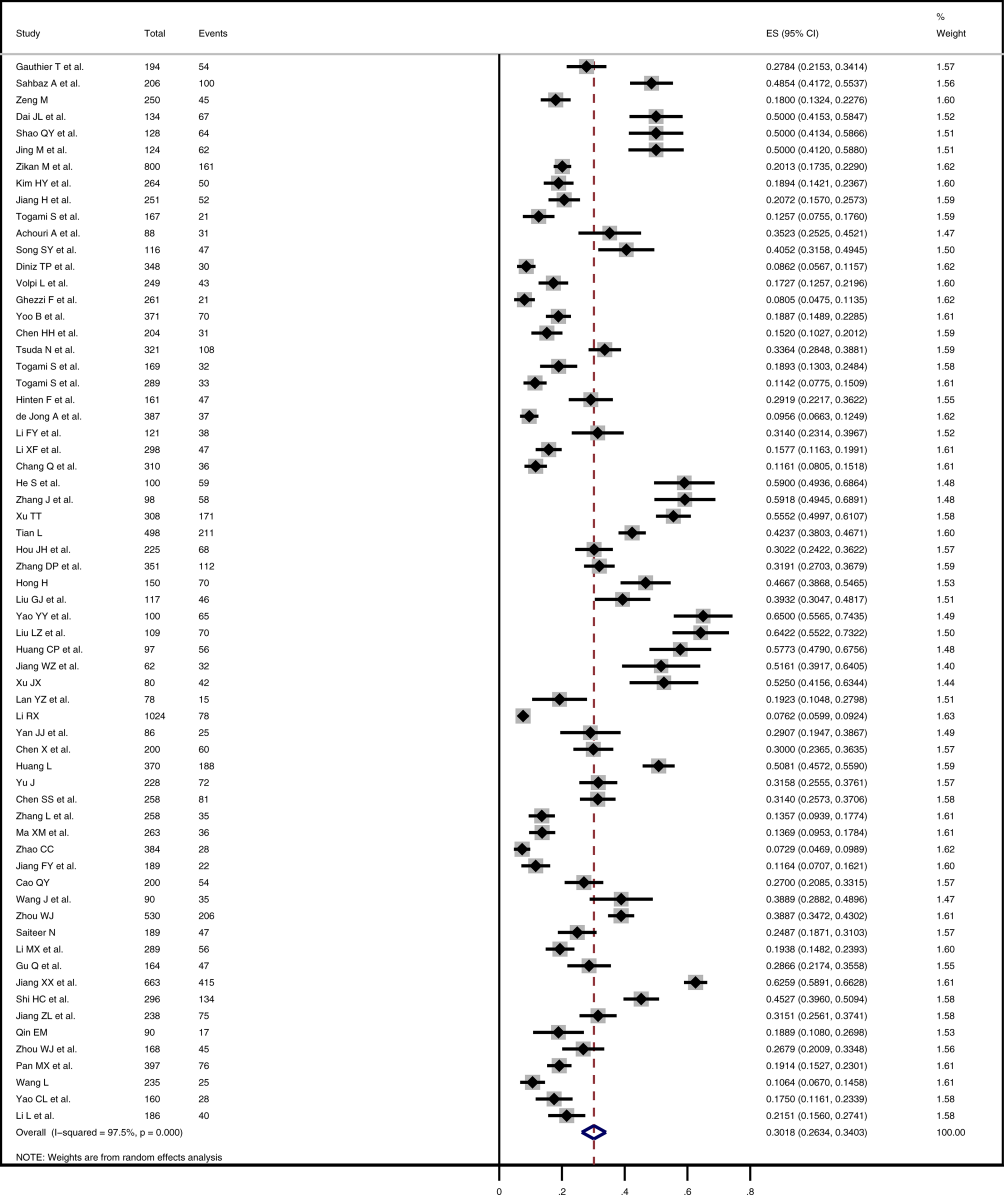
Forest plot of lymphocele incidence.

**Figure 4:**
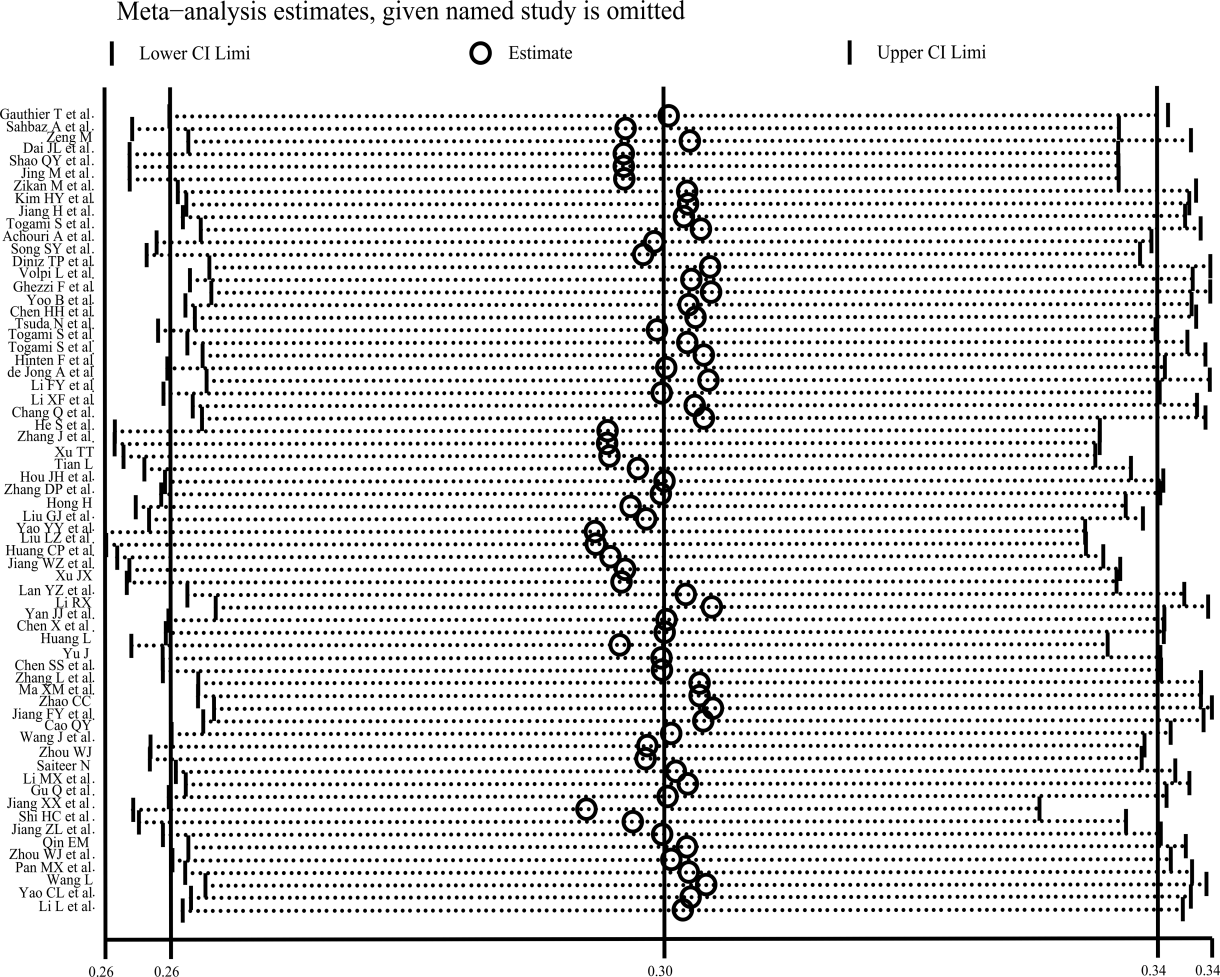
Sensitivity analysis of lymphocele incidence.

Among 15,739 patients with gynecological malignancies who underwent PLND, 4,327 were diagnosed with lymphocele. As shown in **Figure 3**, the incidence rates in individual studies ranged from 7.29% to 65.00%, with a pooled incidence of 30.18% (95% CI: 26.34%–34.03%). Sensitivity analysis indicated that the results were relatively stable (**Figure 4**). The study with the largest sample size was a retrospective cohort study from China (51), which included 1,024 participants and reported a lymphocele incidence of 7.62%. This was followed by a prospective cohort study from the Czech Republic (15), which included 800 participants and reported an incidence of 20.13%. Differences in surgical strategies, study design and postoperative detection methods may partly account for the varied incidence of lymphocele across studies.

#### 3.3.2 Preoperative clinical characteristics

Sixteen studies analyzed the association between advanced age (>50 years) and lymphocele development, with low between-study heterogeneity (I² = 29.1%, P = 0.132). Pooled results from the fixed-effects model revealed that age >50 years was a significant risk factor for lymphocele formation (OR = 1.21, 95% CI:1.02-1.42, P = 0.024).

Given the discrepancy in BMI cutoff values between Chinese guidelines and the WHO international standards, a stratified analysis was performed based on different diagnostic criteria. Thirteen studies conducted in China adopted a BMI cutoff of ≥24 kg/m² and exhibited moderate heterogeneity (I² = 65.6%, P < 0.001). Random-effects meta-analysis demonstrated that BMI ≥24 kg/m² was associated with an increased risk of lymphocele (OR = 1.45, 95% CI:1.10-1.93, P = 0.009). In contrast, five studies using the WHO cutoff of BMI ≥25 kg/m² showed no significant heterogeneity (I² = 0%, P = 0.658). Fixed-effects pooling indicated that BMI ≥25 kg/m² was not statistically correlated with lymphocele occurrence (OR = 1.18, 95% CI:0.86-1.61, P = 0.308).

Pooled analysis was further performed based on 23 studies regarding diabetes and 18 studies regarding hypertension. The results showed that diabetes was a significant risk factor for lymphocele (OR = 1.30, 95% CI:1.11-1.52, P = 0.001), whereas hypertension was not significantly associated with lymphocele development (OR = 1.08, 95% CI:0.92-1.27, P = 0.337).Seventeen studies explored the association between a history of abdominal surgery and lymphocele formation, with low heterogeneity across the included studies (I² = 5.3%, P = 0.392). Fixed-effects model analysis confirmed that prior abdominal surgery history was not significantly correlated with lymphocele development (OR = 1.09, 95% CI:0.92-1.29, P = 0.322).

#### 3.3.3 Surgical-related factors

A total of 23 studies explored the impacts of the number and scope of lymph node dissection. Dissection of more than 20 lymph nodes was significantly correlated with lymphocele occurrence (OR = 2.75, 95%CI: 2.16–3.51, P < 0.001; I^2^ = 67.5%, P < 0.001). Concurrent pelvic combined with para-aortic lymph node dissection also increased the risk of lymphocele development (OR = 1.86, 95%CI: 1.42–2.43, P < 0.001; I^2^ = 72.7%, P < 0.001). Twenty-five studies compared laparotomy with laparoscopic surgery. The results showed that patients undergoing laparotomy had a significantly higher risk of postoperative lymphocele (OR = 2.76, 95%CI: 2.12-3.58, P < 0.001), with high between-study heterogeneity (I² = 57.5%, P < 0.001). Potential confounding factors, such as the number and extent of lymph node dissection, may exist between the two surgical approaches. Seven studies investigated the correlation between peritoneum closure and lymphoceles. Moderate to high heterogeneity was observed across included studies (I^2^ = 54.6%, P = 0.040), and pooled results indicated that peritoneum closure was significantly associated with lymphocele formation (OR = 2.44, 95%CI: 1.67-3.57, P < 0.001).

Seven studies evaluated the correlation between routine drainage placement and lymphocele occurrence. The pooled data revealed that routine drainage placement was not statistically correlated with lymphocele occurrence (OR = 0.99, 95%CI: 0.55-1.77, P = 0.963; I^2^ = 62.8%, P = 0.013). Further subgroup analyses found that drainage duration over 3 days (OR = 1.60, 95%CI: 1.03-2.46, P = 0.035) and 24-hour postoperative drainage volume exceeding 100 mL (OR = 1.61, 95%CI: 1.10-2.36, P = 0.015) were independent risk factors for lymphoceles. Moreover, seven studies comparing different drainage approaches showed that patients receiving transvaginal drainage had a higher incidence of lymphoceles than those with transabdominal drainage (OR = 2.90, 95%CI: 1.92-4.40, P<0.001; I^2^ = 59.1%, P = 0.023).

Analysis of other surgical influencing factors showed that omentectomy (OR = 1.56, 95%CI: 1.18-2.07, P = 0.002), iliac circumflex lymphadenectomy (OR = 2.56, 95%CI: 1.61-4.07, P < 0.001), and operative duration longer than 3 hours (OR = 1.56, 95%CI: 1.10-2.21, P = 0.013) could markedly elevate the risk of lymphocele formation. Intraoperative use of monopolar electrocautery was linked to a higher lymphocele rate relative to ultrasonic scalpel application (OR = 2.48, 95%CI: 1.75-3.51, P < 0.001). In contrast, lymphatic ligation (OR = 0.64, 95%CI: 0.21-1.91, P = 0.421) and intraoperative blood loss over 200 mL (OR = 1.04, 95%CI: 0.83-1.31, P = 0.706) exhibited no significant statistical association with lymphocele development. Given the limited number of included studies for the above indicators, these findings need to be further validated by high-quality clinical trials in the future.

#### 3.3.4 Tumor pathology-related factors

Sixteen studies compared the risk of postoperative lymphocele across ovarian, endometrial, and cervical cancers. Pairwise analyses showed that cervical cancer (OR = 0.69, 95% CI: 0.48-0.99, P = 0.042) and endometrial cancer (OR = 0.59, 95% CI: 0.40-0.87, P = 0.008) were associated with a significantly lower lymphocele risk compared with ovarian cancer. No significant difference was observed between cervical and endometrial cancer (OR = 1.16, 95% CI: 0.88-1.53, P = 0.286).

Forty-one studies explored the association between FIGO stage and lymphocele incidence. Patients with stage I disease had a lower lymphocele risk than those with stage II disease (OR = 0.49, 95% CI: 0.30-0.80, P = 0.004). Similarly, early-stage disease (stage I+II) was associated with a lower risk than advanced-stage disease (stage III+IV) (OR = 0.51, 95% CI: 0.29-0.91, P = 0.022). Subgroup analysis further demonstrated that stage Ib patients had a significantly lower risk than stage IIa patients (OR = 0.44, 95% CI: 0.32-0.61, P < 0.001).

A total of 26 studies evaluated the impact of histological type on lymphocele, with inconsistent findings observed across subgroups. Six studies compared squamous cell carcinoma with non-squamous carcinoma (including adenocarcinoma and other subtypes). No significant inter-study heterogeneity was found (I^2^ = 0%, P = 0.688). Fixed-effects pooling indicated that squamous cell carcinoma was associated with a higher lymphocele risk (OR = 1.50, 95% CI: 1.01-2.23, P = 0.047). However, 20 studies performing a direct comparison between squamous cell carcinoma and adenocarcinoma showed no heterogeneity (I^2^ = 0%, P = 0.958) and revealed no significant difference in lymphocele risk (OR = 1.06, 95% CI: 0.88-1.27, P = 0.560). These divergent results suggest that the correlation between histological type and lymphocele varies according to grouping criteria and cannot be generalized.

Tumor grade analyses also yielded inconsistent outcomes. No significant difference in lymphocele risk was identified between poorly differentiated (G3) and moderately/well-differentiated (G1/G2) tumors (OR = 1.18, 95% CI: 0.92-1.51, P = 0.187). Nevertheless, moderately differentiated (G2) tumors were associated with a higher risk than well-differentiated (G1) tumors (OR = 1.27, 95% CI: 1.04-1.54, P = 0.017). Such conflicting findings indicate no consistent linear association between tumor differentiation grade and lymphocele formation.

A total of 12 studies indicated that lymphovascular invasion was significantly associated with lymphocele formation (I^2^ = 0%, P = 0.679; OR = 1.25, 95% CI: 1.03–1.52, P = 0.025). Eight studies demonstrated that myometrial invasion depth exceeding 1/2 increased the risk of lymphocele, with moderate between-study heterogeneity (I^2^ = 58.5%, P = 0.018; OR = 1.89, 95% CI: 1.25–2.87, P = 0.003).Twenty studies revealed that positive lymph node status was significantly correlated with an elevated risk of lymphocele, presenting high heterogeneity across included studies (I^2^ = 55.8%, P = 0.001; OR = 1.59, 95% CI: 1.26–2.00, P < 0.001).

#### 3.3.5 Postoperative nutrition and metabolism

Ten studies reported the correlation between postoperative anemia and lymphocele occurrence, with no heterogeneity across this subgroup (I² = 0%, P = 0.655); the fixed-effects model analysis showed that postoperative anemia was associated with an increased risk of lymphocele (OR = 1.20, 95%CI: 1.02-1.40, P = 0.028). Eight studies explored the correlation between postoperative hypoalbuminemia and lymphocele risk, with significant heterogeneity detected (I² = 71.5%, P = 0.001); the random-effects model analysis confirmed that postoperative hypoalbuminemia was a significant risk factor for lymphocele (OR = 1.58, 95%CI: 1.07-2.34, P = 0.023). In addition, 3 studies found no significant association between elevated postoperative triglyceride level (>1.8 mmol/l) and lymphocele development (OR = 1.51, 95%CI: 0.86-2.65, P = 0.154).

#### 3.3.6 Perioperative adjuvant therapies

In the meta-analysis on the association between perioperative adjuvant therapy and lymphocele, significant statistical correlations were identified between postoperative chemotherapy (OR = 1.85, 95% CI: 1.29-2.65, P = 0.001), postoperative concurrent chemoradiotherapy (OR = 2.49, 95% CI: 1.68-3.69, P < 0.001) and lymphocele formation. By contrast, no obvious statistical correlations were observed for preoperative chemotherapy (OR = 1.23, 95% CI: 0.72-2.09, P = 0.453) and postoperative radiotherapy alone (OR = 1.11, 95% CI: 0.77-1.60, P = 0.566).

### 3.4 Sensitivity analysis and publication bias

Sensitivity analyses were conducted to verify the stability and reliability of the pooled results. For risk factors with substantial heterogeneity (I² > 50%) and included more than 2 studies, the one-by-one elimination method was performed, whereby single studies were sequentially excluded to re-estimate the pooled effect sizes. Additionally, sensitivity analysis was also carried out by switching between random-effects and fixed-effects models for all extracted risk factors, and the results were summarized in **Supplementary Material Table 3**.

Among all assessed factors, five variables showed unstable pooled results, namely 24-hour postoperative drainage volume > 100 ml, postoperative drainage duration > 3 days, tumor type (cervical cancer [CC] vs. ovarian cancer [OC]), FIGO stage (stage I+II vs. stage III+IV), and postoperative hypoalbuminemia. Specifically, the synthetic results of the remaining studies became statistically non-significant after excluding certain individual studies, indicating that the conclusions of these five factors were not robust and lacked stability.

In contrast, for all other remaining risk factors, no significant changes were detected in the pooled effect sizes, 95% confidence intervals or P-values after sequential exclusion of any single study. Furthermore, the odd ratios (ORs) and their corresponding 95% confidence intervals obtained from random-effects and fixed-effects models were highly consistent across all included factors. Collectively, these findings confirm that the pooled results of the remaining factors are highly robust and credible, and the overall conclusions of this meta-analysis are stable.

**Figure 5:**
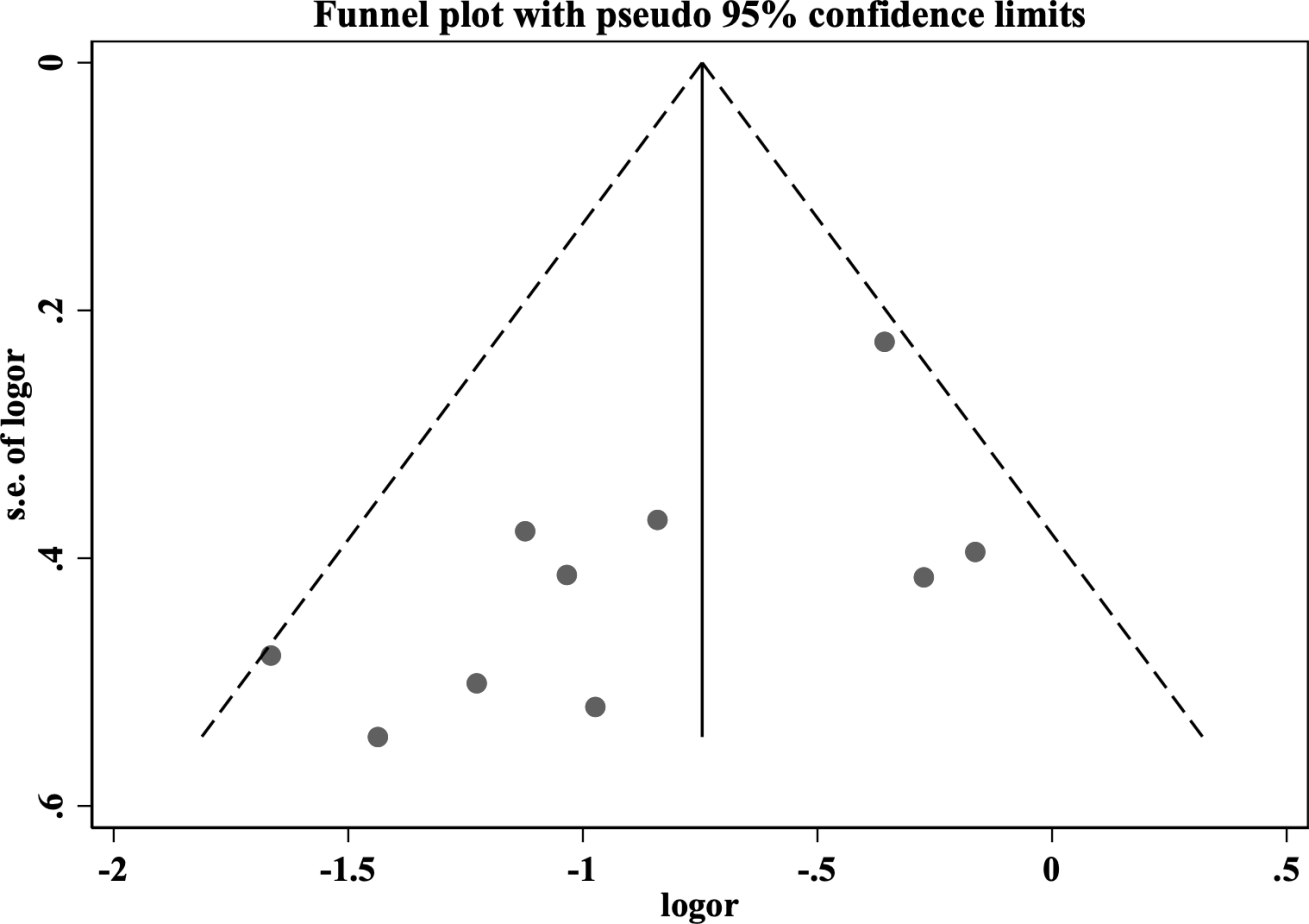
Funnel plot of the correlation between FIGO stage (Stage Ib vs. Stage IIa) and lymphocele.

Egger’s regression test was performed to evaluate potential publication bias for all risk factors with no less than 10 included studies. The test results demonstrated that only the comparison of FIGO stage (Stage Ib vs. Stage IIa) suggested potential publication bias, with a statistically significant Egger’s test value (P = 0.033). Consistently, the corresponding funnel plot for this subgroup also showed obvious overall asymmetry, as visually presented in **Figure 5**.

For all other remaining risk factors included in the meta-analysis, Egger’s test yielded non-significant P values, indicating no significant publication bias. Meanwhile, the funnel plots of these factors were generally symmetric, further confirming the absence of substantial publication bias. Detailed test results are summarized in **Supplementary Material Table 4**.

## 4 Discussion

Pelvic lymphadenectomy serves as a fundamental surgical approach for accurate staging and radical management of gynecological malignancies (1). Nevertheless, this procedure may contribute to a series of perioperative complications. A prior meta-analysis (79) of 1922 patients with gynecologic cancers revealed that individuals with endometrial cancer who received pelvic lymphadenectomy had a markedly higher risk of lymphatic system complications relative to those without lymph node dissection (RR = 8.39, 95% CI: 4.06–17.33). The reported incidence of postoperative lymphocele varies considerably across clinical investigations (15). A 2017 meta-analysis documented that the pooled incidence of lymphocele following pelvic lymphadenectomy ranged from 23% to 65% (4). Consistent with the wide variation in existing literature, the individual study incidence rates in the present meta-analysis varied from 7.29% to 65.00%, with an overall pooled incidence of 30.18%. Such substantial inter-study heterogeneity is primarily attributable to inconsistent diagnostic protocols across centers, which incorporate physical examination, ultrasonography, CT, and MRI for lymphocele screening (15). Most lymphoceles are asymptomatic and identified incidentally on routine postoperative imaging, while symptomatic patients tend to receive targeted imaging assessment; this discrepancy commonly results in underdiagnosis and relevant data bias. Furthermore, the risk factors associated with postoperative lymphocele remain inconclusive and controversial globally (20). On this basis, the present systematic review and meta-analysis synthesized data from 64 eligible studies conducted across nine countries to explore potential correlative factors of lymphocele after gynecological malignancy surgery, aiming to provide evidence-based insights for optimizing perioperative management and mitigating postoperative lymphocele risk.

In terms of preoperative clinical characteristics, age, body mass index (BMI) and diabetes were statistically correlated with postoperative lymphocele formation, while the correlation intensity was limited with obvious inter-study heterogeneity. The present study identified that age over 50 years was potentially associated with lymphocele development, which was consistent with previous findings in patients undergoing pelvic lymphadenectomy for prostate cancer (80). This trend may be attributed to degenerative changes in lymphatic vessel structure, decreased compensatory capacity of lymphatic collateral circulation, and physiological decline in endogenous estrogen levels in elderly patients, which could impair lymphatic drainage and increase the possibility of lymphatic fluid accumulation (61, 81, 82). The correlation between BMI and lymphocele has long been debatable. Based on the diagnostic criteria for Chinese populations, overweight status (BMI≥24 kg/m²) was significantly correlated with an elevated risk of lymphocele in the pooled analysis. In contrast, no significant correlation was observed in studies adopting the international WHO standard (BMI≥25 kg/m²). Excessive accumulation of abdominopelvic adipose tissue in overweight and obese patients limits intraoperative surgical exposure, increases the difficulty of lymph node dissection and lymphatic vessel closure, and elevates postoperative exudation, which may indirectly increase lymphocele risk (54). Accumulated clinical studies have verified a higher incidence of lymphocele in obese individuals (83), suggesting that weight management may help reduce the risk of symptomatic lymphocele (31). Diabetes mellitus was correlated with lymphocele occurrence. The hyperglycemic microenvironment could impair the chemotaxis, phagocytosis and bactericidal functions of immune cells, thereby hindering postoperative tissue repair and local microenvironment homeostasis (38, 49, 67). Nevertheless, no significant association was found between hypertension and lymphocele in this meta-analysis. Previous studies have reported that a history of abdominal surgery may cause pelvic adhesion, which is linked to prolonged operation time and increased perioperative complications (84). However, such correlation was not confirmed in our analysis, indicating that isolated pelvic adhesion could not serve as an independent influencing factor for lymphocele.

Surgical parameters constitute the core influencing factors for postoperative lymphocele. Our results revealed that more than 20 resected lymph nodes, combined pelvic and para-aortic lymphadenectomy, iliac circumflex lymphadenectomy, omentectomy and retroperitoneal closure were all correlated with lymphocele formation. Lymphatic vessel injury is the key contributor to lymphocele development. A greater number of resected lymph nodes corresponds to a higher risk of lymphatic vessel damage and more residual lymphatic stumps, thereby increasing the risk of postoperative lymphatic leakage (85). Similarly, extensive combined pelvic and para-aortic lymphadenectomy results in numerous broken lymphatic vessels and excessive lymphatic fluid leakage. Moreover, para-aortic lymphadenectomy usually extends to the level of the renal veins, which may injure adjacent lymphatic vessels and lymphatic trunks, block normal lymphatic drainage, and hinder the return of lower extremity and abdominal lymph fluid. The subsequent accumulation of lymphatic fluid in the pelvic cavity is closely related to lymphocele formation, which has been validated by multiple studies (86–88). Concurrent iliac circumflex lymphadenectomy and omentectomy may increase lymphocele risk, as previously confirmed in relevant studies on lymphedema (89, 90). Preservation of iliac circumflex lymph nodes helps protect fine lymphatic vessels and collateral circulation pathways, thereby reducing lymphocele incidence (91, 92). The omentum possesses abundant fenestrated capillaries that facilitate the transportation and absorption of interstitial fluid and macromolecular substances; therefore, omentoplasty exerts a preventive effect against lymphocele formation (93). Meanwhile, maintaining an open retroperitoneum intraoperatively promotes the absorption of lymphatic fluid by the peritoneum and omentum, and reduces the risk of pelvic fluid accumulation and secondary infection (8, 94). On the contrary, retroperitoneal closure is associated with a higher incidence of lymphocele (94, 95). Adequate ligation of lymphatic stumps during surgery has been proven to effectively reduce lymphocele occurrence, shorten hospital stay, alleviate patient discomfort, lower infection risk, and ensure timely delivery of adjuvant therapy (96). However, our study failed to confirm the correlation between lymphatic ligation and lymphocele formation, which may be attributable to the limited number of included studies.

Accordingly, clinicians should fully evaluate patients’ general conditions before surgery, clarify tumor stage and lymph node metastasis based on imaging examinations, and avoid unnecessary extensive lymphadenectomy while ensuring radical oncological efficacy. During lymphadenectomy, delicate surgical manipulation, routine preservation of an open retroperitoneum, complete ligation of lymphatic stumps, and minimized injury to adipose tissue and surrounding lymphatic vessels are highly recommended. In recent years, sentinel lymph node (SLN) biopsy has attracted increasing attention in the management of early-stage gynecological malignancies. Negative SLN biopsy indicates a low probability of lymph node metastasis, which helps reduce unnecessary routine pelvic lymphadenectomy and narrow the surgical scope. At present, this technique has been confirmed to achieve reliable long-term oncological safety in low-risk endometrial cancer. Multiple studies have demonstrated that SLN biopsy independently reduces postoperative lymphocele risk compared with systematic pelvic lymphadenectomy (21, 29, 54, 97). Nevertheless, SLN biopsy alone has not been widely recognized as a standard strategy for cervical cancer and high-risk endometrial cancer, due to the lack of definitive evidence from prospective randomized controlled trials, and its oncological safety remains to be further verified (98, 99).

The association between surgical approach and lymphocele formation remains controversial. Our study found that patients undergoing laparotomy presented a significantly higher lymphocele risk than those receiving laparoscopic surgery, which was consistent with the findings of a 2023 meta-analysis (86). A large-scale GOG trial demonstrated that although the incidence of intraoperative complications was comparable between open and laparoscopic lymphadenectomy, patients undergoing laparotomy experienced more postoperative adverse events and longer hospital stay (100). However, one study found no significant difference in postoperative lymphocele incidence between open and robotic-assisted laparoscopic pelvic lymphadenectomy (80). The selection of intraoperative energy devices is closely associated with lymphocele development. Accumulated studies have shown that ultrasonic scalpels are superior to electrocoagulation in reducing lymphatic leakage and shortening postoperative drainage time (101). Our results indicated that the application of monopolar electrosurgery was linked to a higher lymphocele risk relative to ultrasonic scalpels. Monopolar electrocoagulation can only coagulate tiny lymphatic vessels with limited sealing efficacy for large lymphatic trunks. In addition, the eschar formed by monopolar coagulation is prone to shedding after surgery, which may reopen previously sealed lymphatic vessels and increase lymphatic fluid leakage. In contrast, ultrasonic scalpels cause limited thermal damage, enable precise tissue dissection and reliable lymphatic vessel closure, and thus effectively reduce lymphocele susceptibility (102).

Accumulated evidence has indicated that prolonged operative duration is correlated with lymphatic complications. Benito et al. (103) and Saemathong et al. (104) reported that operation time exceeding 250 minutes and 4 hours served as independent risk factors for lymphocele and lymphedema, respectively. Our findings drew a similar conclusion that operative duration longer than 3 hours was correlated with increased lymphocele risk. However, the association between intraoperative blood loss and lymphocele remains inconclusive. Benito et al. (103) argued that intraoperative blood loss over 200 mL significantly increased the risk of postoperative lymphatic complications, whereas no such statistical correlation was identified in our study, which was consistent with the results reported by Tsuda et al. (27). Such inter-study discrepancies may be attributed to diversified intraoperative hemostatic strategies, including lymphatic ligation and bipolar vessel sealing devices. Further high-quality prospective studies are required to verify the true correlation between blood loss and lymphocele and eliminate the interference of relevant confounding factors.

No significant correlation was observed between routine drainage placement and lymphocele incidence in our study, which was consistent with the research results released by the European Organization for Research and Treatment of Cancer-Gynecologic Cancer Group (EORTC-GCG) (105). Several studies further pointed out that indwelling drainage could not prevent lymphocele formation; on the contrary, when the pelvic peritoneum was kept open, drainage placement might increase the risk of symptomatic lymphocele (106). In addition, our study identified that drainage duration longer than 3 days and 24-hour postoperative drainage volume over 100 mL were closely correlated with elevated lymphocele risk. Long-term indwelling drainage induces local inflammatory responses, delays wound healing and lymphatic stump closure, disturbs lymphatic fluid reflux and reabsorption, and ultimately increases lymphocele formation risk (2). Therefore, drainage tubes should be removed within 3 days if the patient’s physical condition permits, so as to reduce pelvic lymphocele incidence (107, 108). Moreover, our study observed that patients receiving transvaginal drainage had a higher lymphocele incidence than those with transabdominal drainage, which may be related to insufficient drainage and increased risk of secondary infection. However, this conclusion was limited by the small number of relevant studies and needs further verification.

In terms of tumor pathological features, tumor type, clinical stage, lymphovascular invasion, myometrial invasion depth and lymph node positivity were all correlated with postoperative lymphocele formation to varying degrees. Different studies have reported inconsistent high-risk tumor types for lymphocele (15, 16). Our study confirmed that ovarian cancer was associated with a higher lymphocele risk compared with cervical cancer and endometrial cancer. This discrepancy may be explained by the greater complexity of ovarian cancer surgery. Ovarian cancer usually requires extensive surgical resection including omentectomy and partial peritoneal excision, which may weaken the peritoneal capacity for excessive lymphatic fluid absorption (109). Patients with advanced tumor stages present extensive cancer infiltration, which inevitably requires expanded lymphadenectomy. In the presence of deep myometrial invasion and lymphovascular invasion, the overall surgical scope is further enlarged, leading to increased secretion of lymphatic fluid and tissue exudate (53). In addition, metastatic lymph nodes are often densely adhered to surrounding blood vessels and nerves, increasing the difficulty of surgical dissection and the risk of injury to adjacent lymphatic tissues, which may further elevate lymphocele incidence (54). Such findings have been verified in multiple studies (110, 111). Nevertheless, subgroup analyses on histological type and tumor grade failed to obtain consistent conclusions in our study, and relevant correlations still require further research for confirmation.

In terms of postoperative nutrition and metabolism, our study found that patients with postoperative anemia and hypoalbuminemia presented an increased risk of lymphocele. Existing studies have demonstrated that decreased albumin and hemoglobin levels reduce plasma colloid osmotic pressure, promote extravasation of intravascular fluid into the interstitial space, and result in excessive local fluid accumulation, which is involved in lymphocele formation (112). In addition, low hemoglobin levels reduce blood oxygen-carrying capacity. Tissue hypoxia impairs the functional activity of immune cells, weakens their abilities in pathogen phagocytosis, antibody production and cytokine secretion, delays tissue repair and increases the risk of secondary infection (113). Therefore, close attention should be paid to postoperative anemia and hypoalbuminemia, and timely supportive treatment should be administered to reduce lymphocele occurrence and relevant infectious complications.

In the aspect of perioperative adjuvant therapy, postoperative chemotherapy and postoperative concurrent chemoradiotherapy were significantly correlated with higher lymphocele risk. Postoperative adjuvant chemotherapy may induce chronic peritoneal inflammation, impair peritoneal reabsorption capacity, disrupt the dynamic balance of tissue exudation and absorption, and thus contribute to pelvic lymphocele formation (75). Moreover, chemotherapy may impair systemic immune function to varying degrees, reduce the body’s resistance to pathogenic microorganisms, and not only increase the susceptibility to pelvic lymphocele, but also elevate the risk of secondary infectious complications (76). Previous studies have indicated that postoperative radiotherapy may cause extensive perilymphatic tissue fibrosis, obstruct lymphatic reflux, inhibit lymphatic proliferation and hinder compensatory lymphatic vessel regeneration. Meanwhile, radiation-induced lymph node fibrosis may weaken lymphatic filtration function, which serves as a potential factor for lymphocele development (114, 115). However, no significant correlation was found between postoperative radiotherapy alone and lymphocele in our study, which was in line with the results of a meta-analysis published in 2023 (86).

This study systematically investigated the risk factors for lymphocele subsequent to lymphadenectomy in patients with gynecological malignancies, offering evidence-based references for individualized perioperative management and high-risk population screening. Notwithstanding, several inherent limitations of the present analysis should be noted. Potential bias sources are summarized as follows. First, the inclusion of only English and Chinese literature may introduce language bias. Most eligible studies were single-center investigations conducted in China, leading to unbalanced regional distribution and limited global representativeness. Regional heterogeneity in diagnostic criteria, postoperative follow-up regimens and imaging surveillance protocols may affect the stability of pooled results and restrict the external generalizability of our findings. Furthermore, the included studies were predominantly retrospective observational studies, which are inherently prone to selection bias, information bias and unmeasured confounding variables, thereby weakening the overall evidence quality.

In addition to the aforementioned bias issues, several other limitations remain. First, although common perioperative indicators were comprehensively extracted, several clinically important factors, such as perioperative anticoagulation strategies (e.g., low-molecular-weight heparin use), could not be quantitatively synthesized due to insufficient and inconsistent raw data, reflecting suboptimal standardization of data recording in current clinical practice. Secondly, this analysis only focused on the overall incidence of lymphocele, without conducting stratified comparisons of clinically meaningful subtypes, including symptomatic, complicated and infected lymphoceles. Further rigorous stratification analyses targeting disease severity and clinical phenotypes are needed to refine risk assessment systems and facilitate targeted intervention strategies. Large-scale, multicenter prospective studies with unified diagnostic and evaluation criteria are urgently required to validate our findings and promote clinical extrapolation.

## 5 Conclusion

This meta-analysis systematically analyzed 64 eligible studies involving 15,739 patients with gynecological malignancies, revealing that the pooled overall incidence of postoperative lymphocele after pelvic lymphadenectomy was 30.18%. We comprehensively identified and categorized the relevant risk factors for postoperative lymphocele into five major dimensions: preoperative clinical characteristics, surgical factors, tumor pathological factors, postoperative nutrition and metabolism, and perioperative adjuvant therapy.

Specifically, in terms of preoperative characteristics, age >50 years, BMI ≥24 kg/m² (Chinese criteria), and diabetes were associated with an increased risk of lymphocele. For surgical factors, laparotomy, combined pelvic and para-aortic lymphadenectomy, resection of more than 20 lymph nodes, retroperitoneal closure, omentectomy, application of monopolar electrosurgery, prolonged operation time (>3 hours), transvaginal drainage, 24-hour drainage volume >100 ml, and prolonged drainage were all significantly associated with elevated lymphocele incidence. Regarding tumor pathology, ovarian cancer, advanced FIGO stage, lymphovascular invasion, deep myometrial invasion, and lymph node positivity were closely related to lymphocele formation. In terms of postoperative nutrition, anemia and hypoalbuminemia also increased the risk. For adjuvant therapy, postoperative chemotherapy and concurrent chemoradiotherapy were associated with lymphocele, whereas radiotherapy alone showed no significant association.

**Figure 6:**
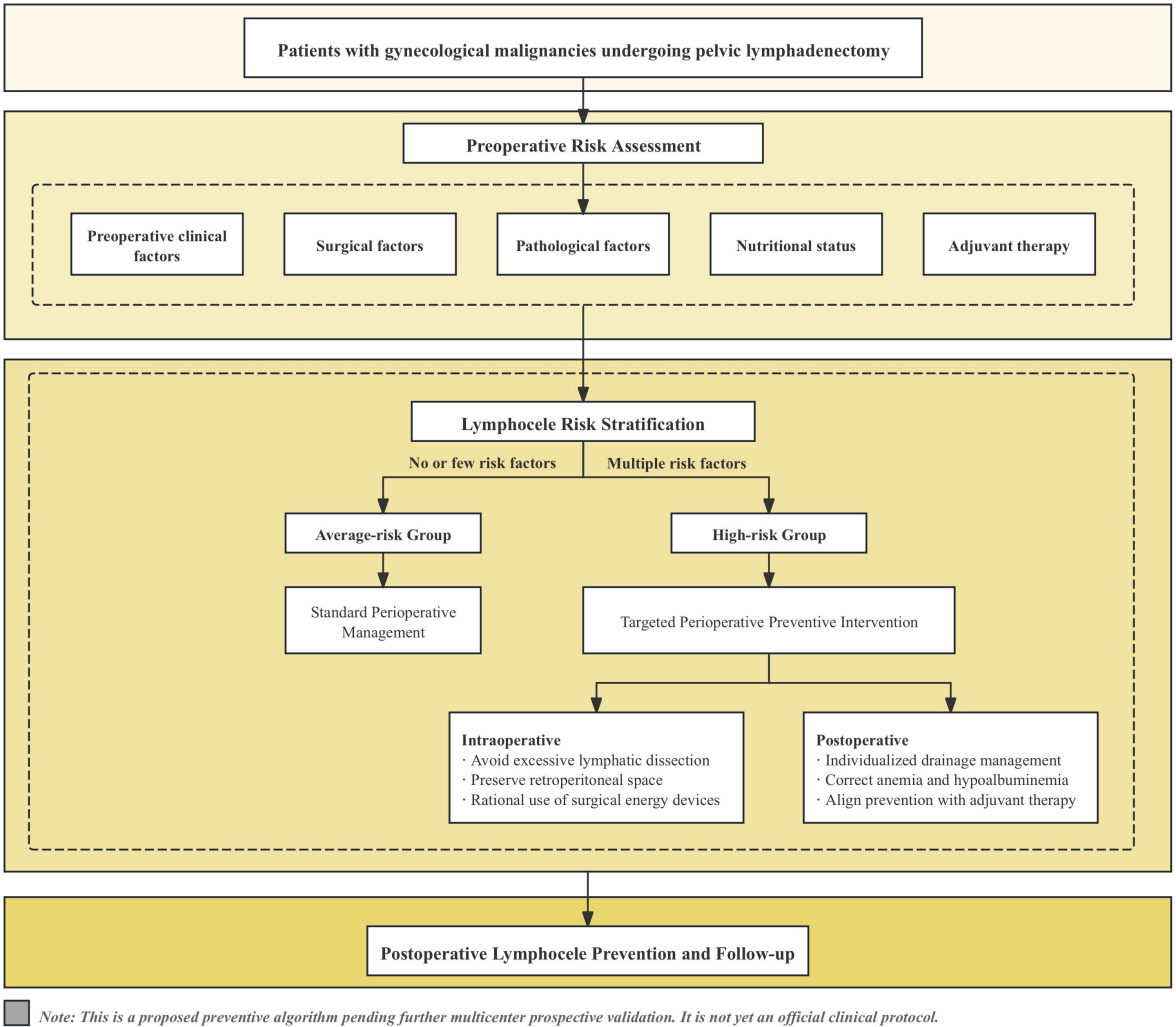
Flowchart of the risk-stratified algorithm for the individualized prevention of postoperative lymphocele in patients with gynecological malignancies. This proposed preventive algorithm is a preliminary framework derived from meta-analytic evidence, pending further multicenter prospective validation. It has not yet been formally sanctioned as a standardized clinical protocol.

Based on the risk factors identified in this study, clinicians can preoperatively assess lymphocele risk by integrating patients’ baseline status, surgical approach, tumor pathology, and adjuvant treatment plans. This enables a stratified screening and individualized management framework that distinguishes average-risk from high-risk populations, guiding differentiated perioperative interventions. The logical workflow of this risk-stratified decision-making algorithm is illustrated in **Figure 6**. Patients without significant risk factors may receive standard perioperative care, whereas those with multiple risk factors require targeted, continuous intervention.

Intraoperatively, oncological radicality should be maintained while avoiding unnecessary extensive lymphadenectomy, keeping the retroperitoneal space open when feasible, and selecting appropriate energy devices to reduce iatrogenic lymphatic injury. Postoperatively, drainage tubes should be removed promptly based on dynamic output changes, and nutritional abnormalities such as hypoalbuminemia and anemia should be actively corrected. Comprehensive preventive measures should also be aligned with adjuvant treatment regimens to effectively lower lymphocele risk. Notably, this risk-stratified individualized model is a preliminary theoretical framework derived from meta-analytic findings; it awaits further validation via large multicenter prospective studies and is not yet endorsed as an official standardized clinical protocol. At present, it serves only as a reference for personalized clinical decision-making and enhances the translational value of our findings.

Nevertheless, several limitations should be acknowledged. Inconsistent findings exist regarding risk factors including age, body mass index, and anemia. Moreover, most included studies were single-center and retrospective, introducing clinical heterogeneity. Well-designed, multicenter prospective studies with uniform diagnostic criteria are therefore warranted to further validate risk factors, optimize high-risk screening, and refine standardized, individualized prevention and management strategies for postoperative lymphocele in gynecologic oncology.

## Data Availability

The original contributions presented in the study are included in the article/**Supplementary Material**. Further inquiries can be directed to the corresponding author.
