# Supplementary material for: Risk factors associated with postoperative lymphocele in patients with gynecological malignant tumors: a systematic review and meta-analysis
Source: Front Oncol. 2026 Jun 10;16:1828153. doi: 10.3389/fonc.2026.1828153 (PMC13291012; doi:10.3389/fonc.2026.1828153)
Supplement: Supplementary file 1 [file DataSheet1.docx]

Supplementary Material

# 1 Supplementary Tables

**Table 1** Table showing the characteristics of included studies.

| Author | Country | Publication year | Inclusion period | Study design | Cancer | Ages* (years) | Surgical approach | Imaging | Total  follow-up time | Mean follow-up time* | Diagnosis time* | NOS score |
| --- | --- | --- | --- | --- | --- | --- | --- | --- | --- | --- | --- | --- |
| Zikan M et al.(15) | Czech Republic | 2015 | February 2006-November 2010 | Prospective cohort study | EC/OC/CC/VC | 55(19–87) | LT, LS | US | 2 years | 37.2 months | 4.8(0.5−14.5) months | 7 |
| Kim HY et al.(16) | Korea | 2004 | March 1999-February 2003 | Retrospective cohort study | EC/OC/CC/other | NA | NA | US, CT, MRI | 6 months | NA | 1.8 months | 7 |
| Jiang H et al.(17) | Zhejiang, China | 2023 | January 2017-January 2022 | Retrospective cohort study | EC/CC | 57.37±10.00 | LT, LS | US, CT, MRI, X-ray | 3 months | NA | NA | 7 |
| Togami S et al.(18) | Japan | 2020 | April 2007-August 2017 | Retrospective cohort study | CC | 41(20–74) | LT | US, CT | NA | NA | NA | 7 |
| Achouri A et al.(19) | France | 2013 | January 2007-November 2008 | Retrospective cohort study | EC/OC/CC | 61(17–87) | LT, LS | US, CT | NA | 16.3±9.5 months | 3 months | 7 |
| Song SY et al.(20) | Korea | 2020 | March 2013-May 2016 | Retrospective cohort study | EC/OC/CC | 52.9±11.35 | LT, LS | CT, MRI, PET-CT | 2 years | NA | 10.5(6–242) days | 8 |
| Diniz TP et al.(21) | Brazil | 2020 | November 2012-January 2020 | Retrospective cohort study | EC | 60(28–86) | LT, LS | CT, MRI | 2 years | 25.4(2–64) months | 4.7(0.8–22.3) months | 7 |
| Volpi L et al.(22) | Italy | 2019 | March 2010-December 2016 | Retrospective cohort study | EC | 64.5±9.4 | LT, LS, RALS | US | 3 years | 53.2±12.3(11–60) months | NA | 8 |
| Gauthier T et al.(23) | France | 2012 | 2005-2011 | Retrospective case-control study | OC | NA | LT, LS | US, CT | NA | 24.8 (1–74) months | NA | 7 |
| Ghezzi F et al.(24) | Italy | 2012 | March 2002-October 2010 / November 1991-February 2002 | Retrospective cohort study | EC | NA | NA | US | NA | NA | 5 weeks | 7 |
| Yoo B et al.(25) | Korea | 2017 | 2009-2014 | Retrospective cohort study | UC/OC/CC/other | 50.8±11.1 | LT, LS | US, CT, MRI | 2 years | NA | NA | 8 |
| Chen HH et al.(26) | Taiwan, China | 2019 | January 2011-December 2017 | Retrospective cohort study | EC/OC/CC/FTC | 53.3±10.6 | LT, LS | US, CT, MRI | NA | NA | NA | 6 |
| Tsuda N et al.(27) | Japan | 2014 | 2005-2011 | Retrospective cohort study | EC/OC/CC/other | NA | LT | CT | 6 months | NA | NA | 6 |
| Togami S et al.(28) | Japan | 2018 | April 2007-August 2017 | Retrospective cohort study | CC | 46(22–74) | NA | US, CT | NA | NA | 3 months | 7 |
| Togami S et al.(29) | Japan | 2019 | January 2007-December 2017 | Retrospective cohort study | EC | 57(24–79) | LT, LS, RALS | US, CT | 3 years | NA | 3 months | 7 |
| Hinten F et al.(30) | The Netherlands | 2011 | January 1988-June 2009 | Retrospective cohort study | VC | 71(31–92) | LT, LS | US | NA | 50.3(0.1–215) months | NA | 6 |
| de Jong A et al.(31) | The Netherlands | 2023 | January 2008-February 2022 | Retrospective cohort study | EC/CC | 48(36–65) | NA | US, CT | 1 year | 47(23–61) months | 59(17−112) days | 7 |
| Sahbaz A et al.(32) | Turkey | 2015 | January 2011-March 2015 | Retrospective case-control study | EC/OC/CC/other | NA | RALS | US, CT, MRI | NA | NA | NA | 6 |
| Li FY et al.(33) | Zhejiang, China | 2018 | January 2013-December 2017 | Retrospective cohort study | CC | NA | NA | US, CT, MRI | 6 months | NA | NA | 6 |
| Li XF et al.(34) | Henan, China | 2008 | January 2002-June 2008 | Retrospective cohort study | EC/OC/CC | 40.3(26–72) | LT, LS | NA | NA | NA | 7−64 days | 5 |
| Chang Q et al.(35) | Ningxia Hui Autonomous Region, China | 2010 | January 2005-April 2010 | Retrospective cohort study | CC | 27–66 | NA | US | 2 weeks | NA | NA | 6 |
| He S et al.(36) | Sichuan, China | 2020 | January 2018-September 2019 | Retrospective cohort study | EC/OC/CC/other | NA | NA | US | NA | NA | NA | 5 |
| Zhang J et al.(37) | Xinjiang Uygur Autonomous Region, China | 2018 | April 2015-March 2017 | Retrospective cohort study | EC/OC/CC/other | 47.3±4.5 | LS | US | 2 weeks | NA | 29 days | 5 |
| Xu TT (38) | Liaoning, China | 2022 | January 2019-June 2021 | Retrospective cohort study | EC/OC/CC/FTC/other | 52±10.1 | LS | US, CT | 3–30 months | NA | NA | 7 |
| Tian L (39) | Shanxi, China | 2017 | June 2013-June 2016 | Retrospective cohort study | EC/OC/CC | NA | NA | US, CT | 4 weeks | NA | NA | 7 |
| Hou JH et al.(40) | Henan, China | 2023 | August 2017-August 2020 | Retrospective cohort study | EC/OC/CC | 46.85±4.69 | LT, LS | US | 4–8 weeks | NA | NA | 7 |
| Zhang DP et al.(41) | Gansu, China | 2020 | January 2017-January 2019 | Retrospective cohort study | EC/OC/CC | 47.28±12.34 | NA | US, CT, MRI | 4–8 weeks | NA | NA | 7 |
| Hong H (42) | Hebei, China | 2024 | January 2018-September 2023 | Retrospective cohort study | EC/OC/CC | NA | LS | US, CT | 6 months | NA | NA | 7 |
| Liu GJ et al.(43) | Zhejiang, China | 2020 | April 2014-May 2019 | Retrospective cohort study | EC/OC/CC/other | 48.12±4.43 | LT, LS | US, CT | NA | NA | NA | 5 |
| Yao YY et al.(44) | Beijing, China | 2013 | July 2010-June 2011 | Retrospective cohort study | EC/OC/CC/other | 51.2±10.1 | NA | US | 4–6 weeks | NA | 29. 0(5−180) days | 7 |
| Liu LZ et al.(45) | Guangdong, China | 2015 | January 2012-December 2013 | Retrospective cohort study | NA | 52.34±8.54 | LT, LS | NA | NA | NA | NA | 5 |
| Huang CP et al.(46) | Guangdong, China | 2016 | January 2006-March 2014 | Retrospective cohort study | EC/OC/CC/other | 58.27±21.43 | LT, LS | US | 1 year | NA | NA | 6 |
| Jiang WZ et al.(47) | Zhejiang, China | 2019 | January 2016-December 2017 | Retrospective cohort study | CC | NA | NA | NA | NA | NA | NA | 5 |
| Zeng M (48) | Anhui, China | 2013 | January 2006-December 2011 | Retrospective case-control study | CC | 49(22–75) | NA | US | NA | NA | NA | 5 |
| Xu JX (49) | Jiangxi, China | 2021 | January 2017-December 2019 | Retrospective cohort study | CC | 50.23±5.73 | LT, LS | US, CT | NA | NA | NA | 5 |
| Lan YZ et al.(50) | Sichuan, China | 2016 | August 2013-August 2015 | Retrospective cohort study | CC | 46.28±8.937 | LT, LS | US, MRI | 3 months | NA | NA | 6 |
| Li RX (51) | Hubei, China | 2022 | January 2016 -February 2020 | Retrospective cohort study | CC | 49(25–75) | LS | US, CT | NA | NA | 2(0.5−21) months | 7 |
| Yan JJ et al.(52) | Jiangxi, China | 2022 | February 2020-February 2021 | Retrospective cohort study | CC | 55.91±6.37 | LT, LS | US | 1 month | NA | NA | 7 |
| Chen X et al.(53) | Zhejiang, China | 2019 | January 2013-December 2017 | Retrospective cohort study | CC | 46.32±14.81 | NA | US, CT | NA | NA | NA | 6 |
| Huang L (54) | Gansu, China | 2022 | June 2011-June 2021 | Retrospective cohort study | CC | 24–85 | LT, LS, RALS | US, CT, MRI | 3 months– 10 years | NA | NA | 7 |
| Yu J (55) | Liaoning, China | 2013 | October 2008-January 2012 | Retrospective cohort study | CC | 48.6(28–72) | NA | CT | 1–3months | NA | NA | 6 |
| Chen SS et al.(56) | Zhejiang, China | 2020 | 2013-2018 | Retrospective cohort study | CC | 51(26–77) | LT | US, CT | 3–65 months | NA | 7−267 days | 7 |
| Zhang L et al.(57) | Shandong, China | 2020 | August 2014 -August 2019 | Retrospective cohort study | CC | 45.3(31–64) | LS | US | NA | NA | NA | 6 |
| Ma XM et al.(58) | Gansu, China | 2017 | January 2008-December 2013 | Retrospective cohort study | CC | 52.5(28–70) | NA | US | 2 weeks | NA | NA | 7 |
| Zhao CC (59) | Liaoning, China | 2013 | January 2008-January 2010 | Retrospective cohort study | CC | 20–72 | LT, LS | NA | NA | NA | NA | 6 |
| Jiang FY et al.(60) | Jiangxi, China | 2016 | March 2012-August 2015 | Retrospective cohort study | CC | 28–67 | NA | US | 2 weeks | NA | NA | 7 |
| Dai JL et al.(61) | Hebei, China | 2015 | October 2008-June 2014 | Retrospective case-control study | CC | 51.7(29–75) | NA | CT, MRI | NA | NA | NA | 6 |
| Cao QY (62) | Jiangxi, China | 2020 | January 2015-December 2018 | Retrospective cohort study | EC | 53.87±7.87 | LS, RALS | US, CT | NA | NA | 35.11±17.95 days | 6 |
| Wang J et al.(63) | Liaoning, China | 2025 | January 2020-January 2024 | Retrospective cohort study | CC | NA | LT, LS | NA | 2 weeks | NA | NA | 7 |
| Zhou WJ (64) | Shandong, China | 2022 | May 2019-April 2020 | Retrospective cohort study | EC/OC/CC | 53.01±10.28 | LT, LS | US, CT, MRI | 1–3months | NA | 33.21±16.97 days | 7 |
| Saiteer N (65) | Xinjiang Uygur Autonomous Region, China | 2014 | December 2011-October 2013 | Retrospective cohort study | EC/CC | NA | LS | US | 6 months | NA | NA | 7 |
| Li MX et al.(66) | Henan, China | 2024 | September 2019-January 2023 | Retrospective cohort study | CC | NA | LT, LS | US | NA | NA | NA | 6 |
| Gu Q et al.(67) | Jiangsu, China | 2020 | January 2013-December 2017 | Retrospective cohort study | CC | NA | LT, LS | US, CT | 8–68 months | 24months | 54(7−336) days | 7 |
| Shao QY et al.(68) | Henan, China | 2022 | March 2019-February 2021 | Retrospective case-control study | CC | 52.63±2.35 | LT, LS | US | 2 weeks | NA | NA | 7 |
| Jiang XX et al.(69) | Yunnan, China | 2022 | January 2013-December 2015/ January 2019- December 2019 | Retrospective cohort study | CC | NA | LT, LS | US, CT, MRI | 6 months | NA | NA | 7 |
| Shi HC et al.(70) | Hebei, China | 2017 | January 2013-April 2016 | Retrospective cohort study | CC | 28.6±3.7 | NA | CT | NA | NA | NA | 6 |
| Jiang ZL et al.(71) | Guangxi Zhuang Autonomous Region, China | 2022 | March 2018-December 2020 | Retrospective cohort study | EC | 53.21±8.46 | RALS | US, CT | 2 weeks | NA | NA | 7 |
| Qin EM (72) | Henan, China | 2022 | February 2018-August 2020 | Retrospective cohort study | EC | 56.42±5.46 | NA | US, CT | NA | NA | NA | 6 |
| Zhou WJ et al.(73) | Shandong, China | 2022 | May 2019-May 2020 | Retrospective cohort study | EC | 56.16±8.707 | LS | US, CT, MRI | 1–2months | NA | NA | 7 |
| Jing M et al.(74) | Henan, China | 2022 | May 2018-May 2020 | Retrospective case-control study | EC | NA | NA | US, CT | NA | NA | NA | 6 |
| Pan MX et al.(75) | Tianjin, China | 2017 | January 2009-April 2016 | Retrospective cohort study | EC | NA | NA | US, CT, MRI | 3–88 months | 30 months | 44.5(5−710) days | 7 |
| Wang L (76) | Hainan, China | 2018 | January 2007-January 2012 | Retrospective cohort study | CC | 54(32–76) | NA | US | NA | NA | NA | 6 |
| Yao CL et al.(77) | Henan, China | 2021 | June 2018-June 2020 | Retrospective cohort study | CC | 51.26±5.34 | LT, LS | US, CT | 6 months | NA | NA | 7 |
| Li L et al.(78) | Henan, China | 2022 | December 2017-December 2019 | Retrospective cohort study | CC | NA | RALS | US | 6 months | NA | NA | 7 |

*Age (years) /Mean follow-up time/Mean diagnosis time was described as median (range) or mean ± standard deviation (SD) or range according to the original study. Abbreviations: EC: endometrial cancer, CC: cervical cancer, OC: ovarian cancer, VC: vulvar cancer, FTC: fallopian tubal cancer, PLND: pelvic lymph node dissection, PALND: para-aortic lymph node dissection, SNNS: sentinel node navigation surgery, SLN: sentinel lymph node, LT: laparotomy, LS: laparoscopy, RALS: robot-assisted laparoscopy, US: ultrasound, CT: computed tomography, MRI: magnetic resonance imaging, NA: not available, NOS: Newcastle–Ottawa Scale.

**Table 2** Subgroup Meta-Analysis of Risk Factors for Lymphocele Formation.

| **Factors** | **Reference** | **No. of Studies** | **No. of Patients** | **I^2^ (%)** | **I^2^ P Value** | **Statistical Method** | **Analysis Model** | **Pooled OR (95% CI)** | **P Value** |
| --- | --- | --- | --- | --- | --- | --- | --- | --- | --- |
| **Preoperative Clinical Characteristics** | | | | | | | | | |
| **Age at surgery** >50 years | ≦50 years | 16 | 3628 | 29.1 | 0.132 | OR | FE | 1.21 (1.02, 1.42) | 0.024^*^ |
| **BMI** |  |  |  |  |  |  |  |  |  |
| ≧24 kg/m^2^ | <24 kg/m^2^ | 13 | 3246 | 65.6 | 0.000 | OR | RE | 1.45 (1.10, 1.93) | 0.009^*^ |
| ≧25 kg/m^2^ | <25 kg/m^2^ | 5 | 1887 | 0.0 | 0.658 | OR | FE | 1.18 (0.86, 1.61) | 0.308 |
| **Comorbidities** |  |  |  |  |  |  |  |  |  |
| Diabetes Mellitus | No | 23 | 5686 | 30.0 | 0.088 | OR | FE | 1.30 (1.11, 1.52) | 0.001^*^ |
| Hypertension | No | 18 | 4483 | 0.0 | 0.498 | OR | FE | 1.08 (0.92, 1.27) | 0.337 |
| **Surgical History** |  |  |  |  |  |  |  |  |  |
| Previous Abdominal Surgery | No | 17 | 4704 | 5.3 | 0.392 | OR | FE | 1.09 (0.92, 1.29) | 0.322 |
| **Surgical-related factors** | | | | | | | | | |
| **Number of Lymph Nodes Dissected** >20 | ≦20 | 23 | 6566 | 67.5 | 0.000 | OR | RE | 2.75 (2.16, 3.51) | 0.000^*^ |
| **Surgical Approach** |  |  |  |  |  |  |  |  |  |
| Laparotomy | Laparoscopy | 25 | 7228 | 57.5 | 0.000 | OR | RE | 2.76 (2.12, 3.58) | 0.000^*^ |
| **Surgical Procedure** |  |  |  |  |  |  |  |  |  |
| PLND+PALND | PLND | 23 | 6707 | 72.7 | 0.000 | OR | RE | 1.86 (1.42, 2.43) | 0.000^*^ |
| Retroperitoneal Closure | Retroperitoneal Leaving Open | 7 | 2265 | 54.6 | 0.040 | OR | RE | 2.44 (1.67, 3.57) | 0.000^*^ |
| Omentectomy | No | 3 | 904 | 0.0 | 0.567 | OR | FE | 1.56 (1.18, 2.07) | 0.002^*^ |
| Iliac Lymph Node Dissection | No | 4 | 874 | 24.3 | 0.266 | OR | FE | 2.56 (1.61, 4.07) | 0.000^*^ |
| Lymphatic Vessel Ligation | No | 3 | 684 | 77.4 | 0.012 | OR | RE | 0.64 (0.21, 1.91) | 0.421 |
| **Duration of Surgery** >3 h | ≦3 h | 2 | 664 | 0.0 | 0.426 | OR | FE | 1.56 (1.10, 2.21) | 0.013^*^ |
| **Surgical Instrument** |  |  |  |  |  |  |  |  |  |
| Monopolar Electrosurgical Unit | Ultrasonic Scalpel | 4 | 692 | 12.1 | 0.332 | OR | FE | 2.48 (1.75, 3.51) | 0.000^*^ |
| **Intraoperative Blood Loss** >200 ml | ≦200 ml | 6 | 2463 | 0.0 | 0.502 | OR | FE | 1.04 (0.83, 1.31) | 0.706 |
| **Drainage** |  |  |  |  |  |  |  |  |  |
| Drain Placement | No | 7 | 1613 | 62.8 | 0.013 | OR | RE | 0.99 (0.55, 1.77) | 0.963 |
| Duration of Drainage >3 days | ≦3 days | 7 | 1867 | 69.5 | 0.003 | OR | RE | 1.60 (1.03, 2.46) | 0.035^*^ |
| Postoperative 24-hour Drainage Volume >100 ml | ≦100 ml | 6 | 1537 | 61.9 | 0.022 | OR | RE | 1.61 (1.10, 2.36) | 0.015^*^ |
| Transvaginal Drainage | Transabdominal Drainage | 7 | 1685 | 59.1 | 0.023 | OR | RE | 2.90 (1.92, 4.40) | 0.000^*^ |
| **Tumor Pathology-Related Factors** | | | | | | | | | |
| **Tumor Type** |  |  |  |  |  |  |  |  |  |
| CC | OC | 14 | 2532 | 65.2 | 0.000 | OR | RE | 0.69 (0.48, 0.99) | 0.042^*^ |
| EC | OC | 14 | 2174 | 70.5 | 0.000 | OR | RE | 0.59 (0.40, 0.87) | 0.008^*^ |
| CC | EC | 16 | 3403 | 57.0 | 0.003 | OR | RE | 1.16 (0.88, 1.53) | 0.286 |
| **FIGO Stage** |  |  |  |  |  |  |  |  |  |
| Stage I | Stage II | 17 | 3903 | 86.1 | 0.000 | OR | RE | 0.49 (0.30, 0.80) | 0.004^*^ |
| Stage I+II | Stage III+IV | 14 | 3177 | 86.4 | 0.000 | OR | RE | 0.51 (0.29, 0.91) | 0.022^*^ |
| Stage Ib | Stage IIa | 10 | 2117 | 35.4 | 0.125 | OR | FE | 0.47 (0.37, 0.60) | 0.000^*^ |
| **Histological Type** |  |  |  |  |  |  |  |  |  |
| SCC | Adenocarcinoma | 20 | 4974 | 0.0 | 0.958 | OR | FE | 1.06 (0.88, 1.27) | 0.560 |
| SCC | Others | 6 | 1393 | 0.0 | 0.688 | OR | FE | 1.50 (1.01, 2.23) | 0.047^*^ |
| **Tumor Grade** |  |  |  |  |  |  |  |  |  |
| G3 | G1+G2 | 14 | 4429 | 52.8 | 0.01 | OR | RE | 1.18 (0.92, 1.51) | 0.187 |
| G2 | G1 | 13 | 2898 | 35.0 | 0.103 | OR | FE | 1.27 (1.04, 1.54) | 0.017^*^ |
| **Lymphovascular Invasion** | No | 12 | 3317 | 0.0 | 0.679 | OR | FE | 1.25 (1.03, 1.52) | 0.025^*^ |
| **Depth of Myometrial Invasion >1/2** | ≦1/2 | 8 | 1559 | 58.5 | 0.018 | OR | RE | 1.89 (1.25, 2.87) | 0.003^*^ |
| **Lymph Node Positivity** | No | 20 | 7461 | 55.8 | 0.001 | OR | RE | 1.59 (1.26, 2.00) | 0.000^*^ |
| **Postoperative Nutritional and Metabolic Parameters** | | | | | | | | | |
| Postoperative Anemia | No | 10 | 3051 | 0.0 | 0.655 | OR | FE | 1.20 (1.02, 1.40) | 0.028^*^ |
| Postoperative Hypoalbuminemia | No | 8 | 2550 | 71.5 | 0.001 | OR | RE | 1.58 (1.07, 2.34) | 0.023^*^ |
| Postoperative Triglycerides level >1.8 mmol/l | ≦1.8 mmol/l | 3 | 1097 | 68.7 | 0.041 | OR | RE | 1.51 (0.86, 2.65) | 0.154 |
| **Postoperative Nutritional and Metabolic Parameters** | | | | | | | | | |
| Neoadjuvant Chemotherapy | No | 10 | 2487 | 75.9 | 0.000 | OR | RE | 1.23 (0.72, 2.09) | 0.453 |
| Postoperative Chemotherapy | No | 22 | 6177 | 81.5 | 0.000 | OR | RE | 1.85 (1.29, 2.65) | 0.001^*^ |
| Postoperative radiotherapy | No | 24 | 6715 | 82.2 | 0.000 | OR | RE | 1.11 (0.77, 1.60) | 0.566 |
| Postoperative concurrent chemoradiotherapy | No | 7 | 1624 | 53.0 | 0.047 | OR | RE | 2.49 (1.68, 3.69) | 0.000^*^ |

Abbreviations: CI: confidence interval, OR: odds ratio, FE: fixed-effects model, RE: random-effects model. Notes: P values for the test of heterogeneity and the overall effect. * Indicates a statistically significant association (P < 0.05). The P value for Egger's test was not applicable (―) for analyses with a limited number of studies (k=2).

**Table 3** Sensitivity Analysis of Factors Associated with Lymphocele Based on Different Effect Models

| **Factors** | **Reference** | **Fixed-effects model Pooled OR (95% CI)** | **Random-effects model Pooled OR (95% CI)** |
| --- | --- | --- | --- |
| **Age at surgery** >50 years | ≦50 years | 1.21 (1.02, 1.42) | 1.22 (0.99, 1.50) |
| **BMI** | | | |
| ≧24 kg/m2 | <24 kg/m2 | 1.43 (1.22, 1.67) | 1.45 (1.10, 1.93) |
| ≧25 kg/m2 | <25 kg/m2 | 1.18 (0.86, 1.61) | 1.19 (0.86, 1.63) |
| **Diabetes Mellitus** | No | 1.30 (1.11, 1.52) | 1.31 (1.08, 1.59) |
| **Hypertension** | No | 1.08 (0.92, 1.27) | 1.08 (0.92, 1.27) |
| **Previous Abdominal Surgery** | No | 1.09 (0.92, 1.29) | 1.10 (0.92, 1.32) |
|  | | | |
| **Number of Lymph Nodes Dissected >20** | ≦20 | 2.62 (2.30, 2.98) | 2.75 (2.16, 3.51) |
| **Laparotomy** | Laparoscopy | 2.70 (2.31, 3.14) | 2.76 (2.12, 3.58) |
| **PLND+PALND** | PLND | 1.84 (1.61, 2.10) | 1.86 (1.42, 2.43) |
| **Retroperitoneal Closure** | Retroperitoneal Leaving Open | 2.67 (2.09, 3.41) | 2.44 (1.67, 3.57) |
| **Omentectomy** | No | 1.56 (1.18, 2.07) | 1.57 (1.18, 2.08) |
| **Iliac Lymph Node Dissection** | No | 2.56 (1.61, 4.07) | 2.40 (1.37, 4.22) |
| **Lymphatic Vessel Ligation** | No | 0.63 (0.38, 1.05) | 0.64 (0.21, 1.91) |
|  | | | |
| **Duration of Surgery >3 h** | ≦3 h | 1.56 (1.10, 2.21) | 1.55 (1.09, 2.21) |
| **Monopolar Electrosurgical Unit** | Ultrasonic Scalpel | 2.48 (1.75, 3.51) | 2.48 (1.71, 3.60) |
|  | | | |
| **Intraoperative Blood Loss >200 ml** | ≦200 ml | 1.04 (0.83, 1.31) | 1.04 (0.83, 1.30) |
| **Drain Placement** | No | 0.95 (0.69, 1.31) | 0.99 (0.55, 1.77) |
| **Duration of Drainage >3 days** | ≦3 days | 1.31 (1.06, 1.62) | 1.60 (1.03, 2.46) |
| **Transvaginal Drainage** | Transabdominal Drainage | 2.61 (2.07, 3.29) | 2.90 (1.92, 4.40) |
| **Postoperative 24-hour Drainage Volume >100 ml** | ≦100 ml | 1.50 (1.21, 1.87) | 1.61 (1.10, 2.36) |
| **Tumor Type** |  |  |  |
| CC | OC | 0.67 (0.55, 0.81) | 0.69 (0.48, 0.99) |
| EC | OC | 0.53 (0.44, 0.64) | 0.59 (0.40, 0.87) |
| CC | EC | 1.19 (1.01, 1.41) | 1.16 (0.88, 1.53) |
| **FIGO Stage** |  |  |  |
| Stage I | Stage II | 0.47 (0.39, 0.56) | 0.49 (0.30, 0.80) |
| Stage I+II | Stage III+IV | 0.50 (0.41, 0.60) | 0.51 (0.29, 0.91) |
| Stage Ib | Stage IIa | 0.47 (0.37, 0.60) | 0.44 (0.32, 0.61) |
| **Histological Type** |  |  |  |
| SCC | Adenocarcinoma | 1.06 (0.88, 1.27) | 1.04 (0.87, 1.26) |
| SCC | Others | 1.50 (1.01, 2.23) | 1.40 (0.93, 2.10) |
| **Tumor Grade** |  |  |  |
| G3 | G1+G2 | 1.17 (1.01, 1.37) | 1.18 (0.92, 1.51) |
| G2 | G1 | 1.27 (1.04, 1.54) | 1.23 (0.95, 1.60) |
| **Lymphovascular Invasion** | No | 1.25 (1.03, 1.52) | 1.25 (1.03, 1.52) |
| **Depth of Myometrial Invasion >1/2** | ≦1/2 | 1.76 (1.35, 2.29) | 1.89 (1.25, 2.87) |
| **Lymph Node Positivity** | No | 1.56 (1.35, 1.80) | 1.59 (1.26, 2.00) |
| **Postoperative Anemia** | No | 1.20 (1.02, 1.40) | 1.20 (1.02, 1.41) |
| **Postoperative Hypoalbuminemia** | No | 1.65 (1.37, 2.00) | 1.58 (1.07, 2.34) |
| **Postoperative Triglycerides level >1.8 mmol/l** | ≦1.8 mmol/l | 1.39 (1.03, 1.88) | 1.51 (0.86, 2.65) |
| **Neoadjuvant Chemotherapy** | No | 1.40 (1.11, 1.76) | 1.23 (0.72, 2.09) |
| **Postoperative Chemotherapy** | No | 1.88 (1.62, 2.17) | 1.85 (1.29, 2.65) |
| **Postoperative radiotherapy** | No | 1.11 (0.96, 1.27) | 1.11 (0.77, 1.60) |
| **Postoperative concurrent chemoradiotherapy** | No | 2.55 (1.98, 3.29) | 2.49 (1.68, 3.69) |

Abbreviations: EC: endometrial cancer, CC: cervical cancer, OC: ovarian cancer, PLND: pelvic lymph node dissection, PALND: para-aortic lymph node dissection, CI: confidence interval, OR: odds ratio.

**Table 4** Egger ́s test results of publication bias

| **Factors** | **Reference** | **No. of Studies** | **Egger's P** |
| --- | --- | --- | --- |
| Age >50 years | ≦50 years | 16 | 0.954 |
| BMI≧24 kg/m2 | <24 kg/m2 | 13 | 0.709 |
| Diabetes Mellitus | No | 23 | 0.579 |
| Hypertension | No | 18 | 0.552 |
| Previous Abdominal Surgery | No | 17 | 0.556 |
| Laparotomy | Laparoscopy | 25 | 0.992 |
| PLND+PALND | PLND | 23 | 0.876 |
| Number of Lymph Nodes Dissected >20 | ≦20 | 23 | 0.150 |
| CC | EC | 16 | 0.994 |
| CC | OC | 14 | 0.846 |
| EC | OC | 14 | 0.493 |
| Stage I | Stage II | 17 | 0.667 |
| Stage I+II | Stage III+IV | 14 | 0.846 |
| Stage Ib | Stage IIa | 10 | 0.033 |
| SCC | Adenocarcinoma | 20 | 0.222 |
| G3 | G1+G2 | 14 | 0.856 |
| G2 | G1 | 13 | 0.416 |
| Lymphovascular Invasion | No | 12 | 0.878 |
| Lymph Node Positivity | No | 20 | 0.683 |
| Postoperative Anemia | No | 10 | 0.072 |
| Neoadjuvant Chemotherapy | No | 10 | 0.224 |
| Postoperative Chemotherapy | No | 22 | 0.978 |
| Postoperative radiotherapy | No | 24 | 0.899 |

# 2 Searching strategy

**eg. Pubmed**

(TS=(“cervical cancer” OR “Uterine Cervical Neoplasm” OR “Cervix Neoplasm” OR “Cervix Neoplasms” OR “Cervical Neoplasms” OR “Cervical Neoplasm” OR “Cancer of the Uterine Cervix” OR “Cancer of Cervix” OR “Cancer of the Cervix”” OR Cervix Cancer” OR “Uterine Cervical Cancer” OR “Uterine Cervical Cancers” OR “Cervical Cancer” OR “Cervical Cancers”) OR TS=("endometrial cancer" OR "Cancer of Endometrium" OR "Endometrium Cancers" OR "Endometrium Cancer" OR "Cancer of the Endometrium" OR "Carcinoma of Endometrium" OR "Endometrium Carcinoma" OR "Endometrium Carcinomas" OR "Endometrial Cancer" OR "Endometrial Cancers" OR "Endometrial Neoplasm" OR "Endometrial Carcinoma" OR "Endometrial Carcinomas") OR TS=("Ovarian Neoplasm" OR "Ovary Neoplasms" OR "Ovary Neoplasm" OR "Ovary Cancer" OR "Ovary Cancers" OR "Cancer of Ovary" OR "Cancer of the Ovary" OR "Ovarian Cancer" OR "Ovarian Cancers") OR TS=("Gynecologic Oncology" OR "gynecological malignancies" OR "gynecological malignancy"OR "gynecologic malignancies" OR "Gynecological Cancer" OR "gynecologic cancer" OR "Female Genital Neoplasm" OR "Female Genital Neoplasms" OR "Gynecologic Neoplasm" OR "Gynecologic Neoplasms") OR TS=("Uterus Neoplasms" OR "Uterine Neoplasm" OR "Neoplasms, Uterus" OR "Uterus Neoplasm" OR "Cancer of Uterus" OR "Uterus Cancers" OR "Uterine Cancer" OR "Uterine Cancers" OR "Cancer of the Uterus" OR "Uterus Cancer")) AND TS=("surgical treatment" OR "surgery" OR "operative" OR "radical surgery" OR "Postoperative Period" OR "Post Operation" OR "After Surgery" OR "Postsurgical" OR "postoperative" OR "Postoperative Periods" OR "Pelvic and Para-Aortic Lymphadenectomy" OR "pelvic and paraaortic lymphadenectomy" OR "pelvic lymphadenectomy" OR "aortic lymphadenectomy" OR "Para-Aortic Lymphadenectomy" OR "Lymph Node Excision" OR "Lymph Node Excisions" OR "Lymphadenectomy" OR "Lymphadenectomies" OR "Lymph Node Dissection" OR "Lymph Node Dissections") AND TS=("Lymphocyst" OR "lymphocyst formation" OR "pelvic lymphocyst" OR "pelvic lymphocysts" OR "pelvic lymphocele" OR "Lymphocele" OR "Lymphoceles" OR "Lymphocoeles" OR "Cyst, Lymphatic" OR "Cysts, Lymphatic" OR "Lymphatic Cysts" OR "Lymphatic Cyst" OR "lymphatic complications" OR "Pelvic Lymphatic Leak" OR "pelvic lymphocele" OR "lymphorrhea") AND TS=(influencing factors OR influence factors OR associating factors OR associated factors OR relevant factors OR related factors OR factors OR relevance OR predictors OR risk factor OR Risk Factor OR Population at Risk OR Populations at Risk OR Risk Scores OR Risk Score OR Risk Factor Scores OR Risk Factor Score OR Health Correlates)
